# IRES-driven Expression of the Capsid Protein of the Venezuelan Equine Encephalitis Virus TC-83 Vaccine Strain Increases Its Attenuation and Safety

**DOI:** 10.1371/journal.pntd.0002197

**Published:** 2013-05-09

**Authors:** Mathilde Guerbois, Eugenia Volkova, Naomi L. Forrester, Shannan L. Rossi, Ilya Frolov, Scott C. Weaver

**Affiliations:** 1 Institute for Human Infections and Immunity, Sealy Center for Vaccine Development, and Department of Pathology, University of Texas Medical Branch, Galveston, Texas, United States of America; 2 Department of Microbiology and Immunology, University of Texas Medical Branch, Galveston, Texas, United States of America; Colorado State University, United States of America

## Abstract

The live-attenuated TC-83 strain is the only licensed veterinary vaccine available to protect equids against Venezuelan equine encephalitis virus (VEEV) and to protect humans indirectly by preventing equine amplification. However, TC-83 is reactogenic due to its reliance on only two attenuating point mutations and has infected mosquitoes following equine vaccination. To increase its stability and safety, a recombinant TC-83 was previously engineered by placing the expression of the viral structural proteins under the control of the Internal Ribosome Entry Site (IRES) of encephalomyocarditis virus (EMCV), which drives translation inefficiently in insect cells. However, this vaccine candidate was poorly immunogenic. Here we describe a second generation of the recombinant TC-83 in which the subgenomic promoter is maintained and only the capsid protein gene is translated from the IRES. This VEEV/IRES/C vaccine candidate did not infect mosquitoes, was stable in its attenuation phenotype after serial murine passages, and was more attenuated in newborn mice but still as protective as TC-83 against VEEV challenge. Thus, by using the IRES to modulate TC-83 capsid protein expression, we generated a vaccine candidate that combines efficient immunogenicity and efficacy with lower virulence and a reduced potential for spread in nature.

## Author Summary

Venezuelan equine encephalitis virus (VEEV) is transmitted by mosquitoes and widely distributed in Central and South America, causing regular outbreaks in horses and humans. Often misdiagnosed as dengue, VEEV infection in humans can lead to lifelong neurological sequelae and is fatal in up to >80% of equine cases, representing a significant socio-economic burden and constant public health threats for developing countries of Latin America. The only available vaccine, the live-attenuated TC-83 strain, is restricted to veterinary use due to its high reactogenicity in humans and risk for reversion to virulence, which could initiate an epidemic. By using an attenuation approach that allows the modulation of the virus capsid protein expression, we generated a new version of TC-83 that is more attenuated but still induces a protective immune response in mice. Additionally, this new vaccine cannot infect mosquitoes, which prevents the risk of spreading in nature. The attenuation approach we describe can be applied to a lot of other alphaviruses to develop vaccines against diseases regularly emerging and threatening developing countries.

## Introduction

Arboviruses (Arthropod-Borne viruses) comprise a group of viruses transmitted among vertebrates by hematophagous arthropods. They include members of a wide range of viral families, such as *Rhabdoviridae*, *Bunyaviridae*, *Flaviviridae* and *Togaviridae*, with a worldwide distribution. The presence of an arbovirus in a particular area depends on the availability of transmission-competent arthropods, as well as amplifying vertebrates (in particular birds or small mammals) susceptible to virus infection and producing sufficient viremia to maintain transmission cycles. Although mostly restricted to sylvatic, enzootic cycles between reservoir vertebrate hosts (mainly rodents and birds) and arthropod vectors, environmental alterations and continuous changes in human and animal demographics have created factors favorable to arboviral emergence from limited cycles, threatening domestic animals and humans [1]. Thus, arboviral epizootics in animals and/or epidemics in human populations are regularly reported. They have significant socio-economic impacts, and contribute to the maintenance of continuous public-health threats around the world.

Venezuelan equine encephalitis virus (VEEV), a positive-strand RNA arbovirus and member of the *Alphavirus* genus in the *Togaviridae* family, is one of the most pathogenic mosquito-borne viruses circulating in South and Central America [2]. In the VEE antigenic complex of alphaviruses that includes 6 subtypes (I to VI), all VEEV strains are found in antigenic subtype I. In this subtype, VEEV strains occur in 4 different antigenic varieties: IAB and IC strains are called “epizootic” or “epidemic” because they efficiently infect equids and produce sufficient viremia to allow oral infection of mosquitoes, thus facilitating high levels of transmission and amplification. These highly efficient equine-mosquito amplification cycles can generate widespread circulation in agricultural areas, usually resulting in spillovers into humans. Varieties ID and IE include enzootic strains, which are typically avirulent for equids and unable to induce high levels of viremia, although some recent IE strains from outbreaks in Mexico are neurovirulent [3], [4]. However, subtypes ID and IE can cause large numbers of human infections via spillover from their sylvatic cycles [2]. Phylogenetic studies indicate that IAB and IC strains derived from subtype ID progenitors [5]. Experimental studies have linked the emergence of VEEV IAB and IC strains to mutations in the E2 glycoprotein, allowing the virus to replicate more efficiently in equids, resulting in greater exposure and/or increased susceptibility to epizootic vectors [6], [7].

Human VEEV infection typically generates moderate to highly incapacitating flu-like symptoms, and is usually misdiagnosed as dengue, resulting in its neglect. Progression to severe encephalitis is observed in about 14% of cases and ultimately death occurs in less than 1%. Although the incidence of fatal disease is relatively low, the neurovirulence of some VEEV strains can lead to lifelong sequelae [8]. In horses, up to >80% of cases can be fatal [9]. Since the first documented outbreaks in the 1930s, several major epidemics have been reported in many countries in Latin America, including Venezuela, Colombia, Peru, Ecuador, Costa Rica, Nicaragua, Honduras, El Salvador, Guatemala, Panama, Mexico, involving hundreds-of-thousands of human and equine cases [2]. VEEV is also highly infectious by aerosol, and had been developed as a biological weapon [10]. Therefore, it represents a major target for which a vaccine is urgently needed to prevent amplification in equids and to protect against human disease.

Like other alphaviruses, VEEV has a positive-sense, single-strand RNA genome of ca. 11.5 kb [11]. The nonstructural protein genes are translated from genomic RNA via a cap-dependent mechanism but the structural genes are translated from a subgenomic message transcribed from negative strand replicative intermediates. The subgenomic RNA is produced in molar excess compared to the genomic RNA, allowing the production of large amounts of the capsid and envelope glycoproteins needed for virion formation [12].

To date, no VEEV vaccine has been licensed for use in humans. VEEV strain TC-83, a live-attenuated, licensed veterinary vaccine, is used to immunize horses in regions endemic for IAB and IC strains, as well as laboratory workers and military personnel. TC-83 was generated by 83 serial-passages of the Trinidad donkey (TrD) IAB strain in guinea pig heart cells [13], and its attenuation relies on only 2 point mutations 14,15. Because RNA viruses exhibit high mutation rates [16], [17], there is a concern that TC-83 may revert to a wild-type, virulent phenotype and cause potentially fatal disease in vaccinees. TC-83 can also infect mosquitoes, as occurred in 1971 during an equine vaccination campaign to prevent spread of an epidemic [18], and thus could initiate an outbreak. In addition, only 80% of human TC-83 vaccinees seroconvert, and reactogenicity is observed in nearly 40% of immunized individuals [19], [20], [21].

In an effort to improve TC-83 attenuation and safety, particularly regarding its potential to be transmitted by mosquitoes from vaccinated horses, a recombinant TC-83 virus, VEEV/mutSG/IRES, was engineered to eliminate the subgenomic promoter and place the expression of the viral structural proteins under the control of the Internal Ribosome Entry Site (IRES) of encephalomyocarditis virus (EMCV) [22], which functions inefficiently in arthropod cells [23], [24]. In this vaccine candidate, the viral subgenomic promoter was inactivated by the introduction of 13 synonymous mutations, and the EMCV IRES was placed upstream of the structural polyprotein gene open reading frame. The resulting recombinant virus, VEEV/mutSG/IRES/1, exhibited an attenuated phenotype in cell culture and *in vivo* in the mouse model, and was unable to replicate in mosquito cells or in live mosquitoes [22]. However, no neutralizing antibody response was detected in vaccinated NIH Swiss mice, and only partial protection against virulent VEEV challenge was achieved.

To improve the immunogenicity of VEEV/mutSG/IRES/1, we developed a new IRES-based variant of TC-83 in which only the capsid protein is placed under IRES translational control, leaving an intact subgenomic promoter driving the expression of the major antigens, the glycoproteins E1 and E2. This new vaccine candidate showed a similar, highly attenuated profile like the original VEE/mutSG/IRES/1 strain and was also unable to replicate in mosquitoes. However, this second generation of IRES-based vaccine candidate was more immunogenic and induced complete protection against lethal VEEV challenge.

## Materials and Methods

### Ethics statement

This study was carried out in strict accordance with the recommendations in the Guide for the Care and Use of Laboratory Animals of the National Institutes of Health. The protocol was approved by the Institutional Animal Care and Use Committees of the University of Texas Medical Branch or the University of Wisconsin.

### Cell cultures

Vero (African green monkey kidney) and baby hamster kidney (BHK-21) cells were obtained from the American Type Cell Culture (ATCC, Manassas, VA) and maintained at 37°C in Dulbecco's minimal essential medium (DMEM) supplemented with 5% fetal bovine serum (FBS), penicillin and streptomycin (PS). C6/36 *Aedes albopictus* cells (ATCC) were propagated at 29°C in DMEM containing 10% FBS, PS and supplemented with 1% tryptose phosphate broth.

### Construction of recombinant VEEV/IRES plasmids

**Figure 1:**
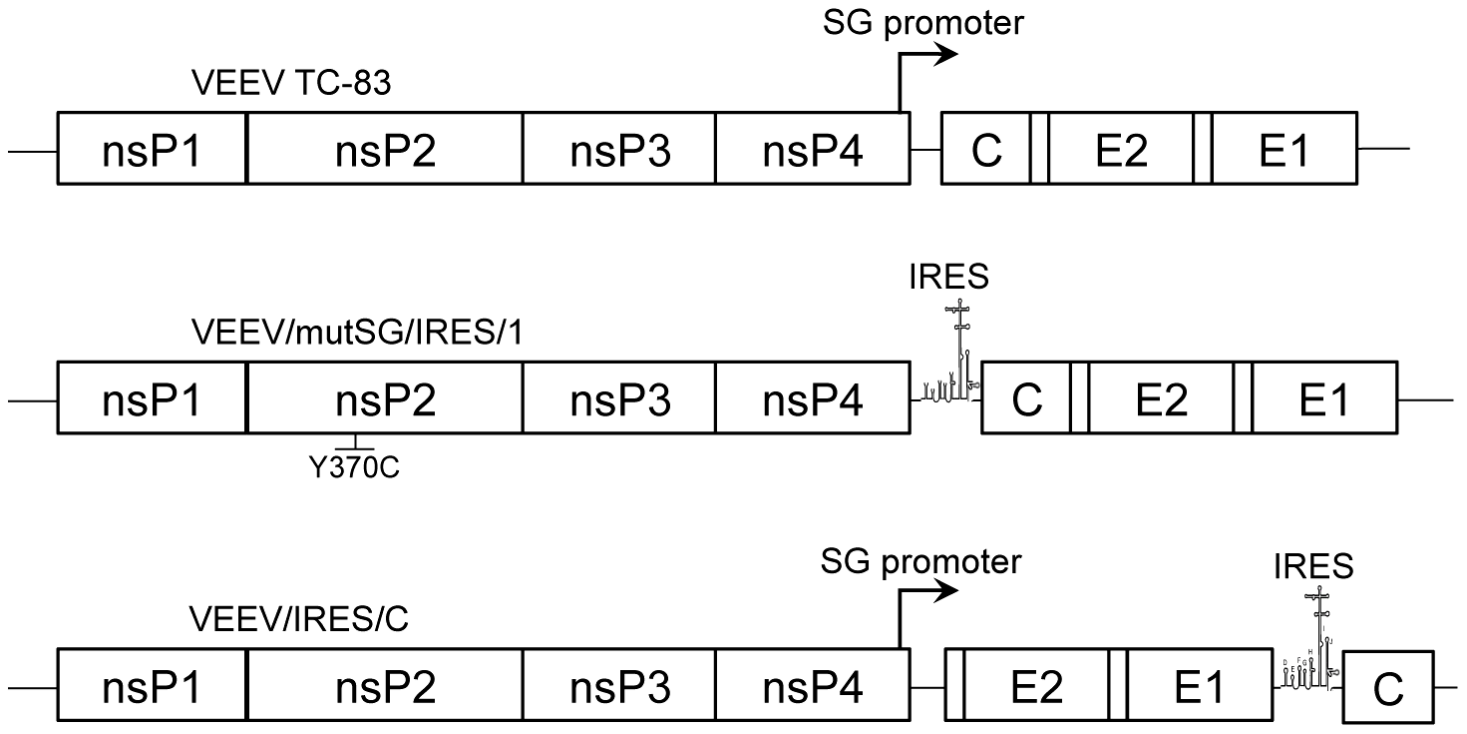
Schematic representation of the recombinant VEEV TC-83/IRES constructs. Alternative positions of the EMCV IRES are indicated. Arrows represent functional subgenomic promoters.

Plasmid pVEEV/mutSG/IRES/1 was described previously [22]. It encodes the complete genome of VEEV strain TC-83, in which the subgenomic promoter is inactivated by 13 synonymous mutations and the structural protein genes are placed under the translational control of the EMCV IRES. The nsP2-coding sequence contains an adaptive mutation that increases replication efficiency. Our new pVEEV/IRES/C strain (Fig. 1) encodes the VEEV TC-83 genome with an active SG promoter. In this plasmid, the capsid gene, under control of the EMCV IRES, was positioned downstream of the E1 gene. This plasmid was constructed using standard PCR-based techniques and details are available from the authors.

### RNA transcription and transfection

After sequencing, a large-scale preparation of pVEEV/IRES/C was obtained using standard methods and purification on CsCl gradients. The plasmid was then linearized with MluI restrictase and subjected to RNA transcription using SP6 RNA polymerase (Ambion, Austin, TX) in the presence of a cap analogue. Each of these steps was analyzed by agarose gel electrophoresis. To rescue virus, *in vitro*-transcribed RNA was transfected into BHK-21 cells by electroporation as previously described [25], [26]. Briefly, one T150 flask of BHK-21 cells was trypsinized, the cells were washed 3 times in PBS, and finally resuspended in 400 µl. One µg of transcribed viral RNA was added to the cells and the mixture was subjected to five pulses at 680v for 99 µsec, at 200 msec intervals. Electroporated cells were resuspended in DMEM containing 10% FBS, seeded into one T75 flask, and incubated at 37°C. When cytopathic effects (CPE) were observed (18-to-24 h post-electroporation), supernatants containing infectious virus were harvested and titrated on Vero cells by plaque assay [27].

### Viral replication

The procedure described above was used to electroporate 4 µg of transcribed RNA into BHK cells. One-fifth of electroporated cells were seeded into 35-mm dishes, and supernatants were harvested at designated time-points post-electroporation and replaced with fresh medium. Alternatively, Vero cells in T25 flasks were infected with a multiplicity of infection (MOI) of 0.1 PFU/cell. After a one-hour incubation at 37°C, cells were washed with PBS and covered with 4 ml of DMEM with 2% FBS. Cell supernatants were collected at different time-points post-infection and replaced with fresh medium. Viruses in harvested supernatants were titrated on Vero cells by plaque assay [27].

### RNA analysis

One-fifth of electroporated BHK cells were seeded into 35-mm dishes and incubated for 4.5 h at 37°C before the supernatant was replaced with 0.8 ml of DMEM supplemented with 1 µg/ml of Actinomycin D and 20 µCi/ml of [^3^H]-uridine. After 4 h of incubation, medium was removed and cells were harvested in 0.8 ml of Trizol (Invitrogen, Carlsbad, CA) for RNA extraction according to manufacturer's protocol. Purified RNA was analyzed by agarose gel electrophoresis after denaturation with glyoxal in dimethyl sulfoxide, as previously described [28]. The gel was then impregnated overnight with 2,5-dipheniloxazol (PPO) and dried. Kodak X-OMAT AR film (Sigma-Aldrich, Saint Louis MO) was exposed to dried gel at −80 °C and autoradiographed.

### Cell culture passages

The VEEV/IRES/C virus was passaged 5 times on Vero cells to determine its genetic stability *in vitro*. Two parallel replicate series were performed at an MOI of 0.1 PFU/cell, and infectious supernatants were harvested 48 h post-infection, titrated by plaque assay and used for the next passage. For viral sequence analysis, RNA was extracted from passage 5 viruses using QIAamp Viral RNA mini kit (Qiagen, Valencia CA) and subjected to 2-step RT-PCR with Superscript III RT System (Invitrogen) and the Phusion DNA polymerase kit (New England BioLabs, Ipswich MA). The resultant 2000 bp amplicons were sequenced using an ABI 3500 Genetic Analyzer (Applied Biosystems, Carlsbad, CA) and alignments and analysis were performed using Sequencher 4.9 software (Ann Arbor, MI).

To assess mosquito cell infectivity, 5 blind serial passages were performed on C6/36 (*Aedes albopictus*) cells seeded in 35-mm dishes and infected at a starting MOI of 1 Vero cell PFU/mosquito cell. After 1 h incubation, inocula were removed, cells were washed 4 times with PBS and 2 ml of DMEM were added. Supernatants were collected 48 h post-infection and 0.4 ml were used to infect C6/36 cells for the next passage. After 5 passages, plaque assays were performed on supernatants from each passage to determine viral titers, as well as RNA extraction and RT-PCR to quantify viral genomes.

### Virus replication in mosquitoes

To evaluate replication competence *in vivo*, we used *Aedes aegypti* mosquitoes from a colony established with individuals collected in Galveston, TX. Five-to-six days post-emergence, mosquitoes were allowed to feed for one hour on an infectious artificial blood meal containing 33% (v/v) defibrinated sheep erythrocytes (Colorado Serum Company, Denver, Co), 33% (v/v) heat-inactivated fetal bovine serum (FBS) (Omega Scientific, Inc., Tarzana, CA) and 33% (v/v) of each individual virus in cell culture medium. The titer of each blood meal was of approximately 5×10^8^ PFU/ml, the highest achievable with Vero cell-derived virus stocks. After feeding, mosquitoes were cold-anesthetized, and engorged individuals were incubated at 27°C with a relative humidity of 70–75% and 10% sucrose *ad libitum* for 10 days. Alternatively, *Ae. aegypti* mosquitoes were injected intrathoracically with ca. 1 µl of a 10^8^ PFU/ml virus stock and incubated as described above.

After 10 days of incubation, mosquitoes were placed individually into 2 ml tubes containing 350 µl of MEM 10% FBS supplemented with 5 µg/ml of Fungizone (Invitrogen) and triturated for 4 min in a Tissue Lyser II (Qiagen, Venlo, Netherlands). Homogenized mosquito samples were centrifuged at 10,000×g for 5 min and 50 µl of supernatants were applied to Vero monolayers in 24-well plates. After incubation for 1 h at 37°C, cells were covered with 1 ml DMEM with 2% FBS and observed for 5 days to detect CPE as signs of infection.

### Virulence, immunogenicity and protection studies

To study virulence, six-day-old CD-1 mice (Charles Rivers, Wilmington, MA) were inoculated intracranially (IC) with 10^6^ PFU of virus in a volume of 20 microliters (µl), or subcutaneously (SC) with 5×10^4^ PFU in a volume of 50 µl. Animals were observed for 2 weeks with daily weight and survival recording. Mice that survived the SC injection were used for immunogenicity and protection studies. Six weeks following initial inoculation with recombinant viruses, blood was collected from the retro-orbital sinus for antibody screening by PRNT as previously described [27], using VEEV TC-83 virus for neutralization. Animals were challenged 3 weeks later with 10^4^ PFU SC of virulent VEEV subtype IC strain 3908 and monitored twice daily for signs of illness, survival and weight loss.

In another experiment, 8-week-old CD-1 mice were vaccinated SC with VEEV strain TC-83 or the IRES-based vaccine candidates at a dose of 10^5^ PFU/mouse, or PBS for unvaccinated controls. Six weeks post-vaccination, animals were challenged SC with 10^4^ PFU of VEEV strain 3908, with daily monitoring for signs of illness, survival and weight loss. Blood samples were collected for 4 days post-vaccination and post-challenge for viremia detection, as well as 5 weeks post-vaccination for antibody measurement by PRNT.

To assess genetic and phenotypic stability of the new IRES-based vaccine candidate *in vivo*, VEEV/IRES/C was subjected to 10 serial, IC passages in six-day-old CD1 mice at a dose of ca. 5×10^4^ PFU per animal. Two parallel passage series were performed (A and B). Animals were euthanized 48 h post-inoculation, and their brain harvested and triturated to determine viral titer by plaque assay. Homogenized brain samples containing the highest titers were used as the inoculum for the next passage in each series. Virulence of the mouse passage 10 (mp10) viruses was compared to the parental strain by inoculating 6-day-old CD1 mice SC with 5×10^4^ PFU, as described above. Stability of the genomic sequences was assessed by RT-PCR on RNA extracted from mp10 viruses and sequencing, as described above. VEEV strains TC-83 and TC-83 mp10A and mp10B, previously described by Kenney *et al.* [29], were included as controls.

### Statistical analyses

All statistical analyses were performed using Prism software (GraphPad version 4.0c, La Jolla, CA). Logrank tests were used to determine significance in survival differences between individual groups. One-way repeated measures ANOVA analyses were performed on the weights of mice following vaccination/challenge. Significance was determined at P<0.05 for all tests.

## Results

### Production of VEEV/IRES/C recombinant virus

This study was designed to develop a VEEV vaccine candidate that would replicate at high titers in vertebrate cells but not in mosquitoes, and that would be immunogenic and protective against lethal VEEV challenge. To evaluate the performances of the new VEEV/IRES/C vaccine candidate compared to the previous IRES-based construct, VEEV/mutSG/IRES/1, we used the latter as a control in this study [22].

**Figure 2:**
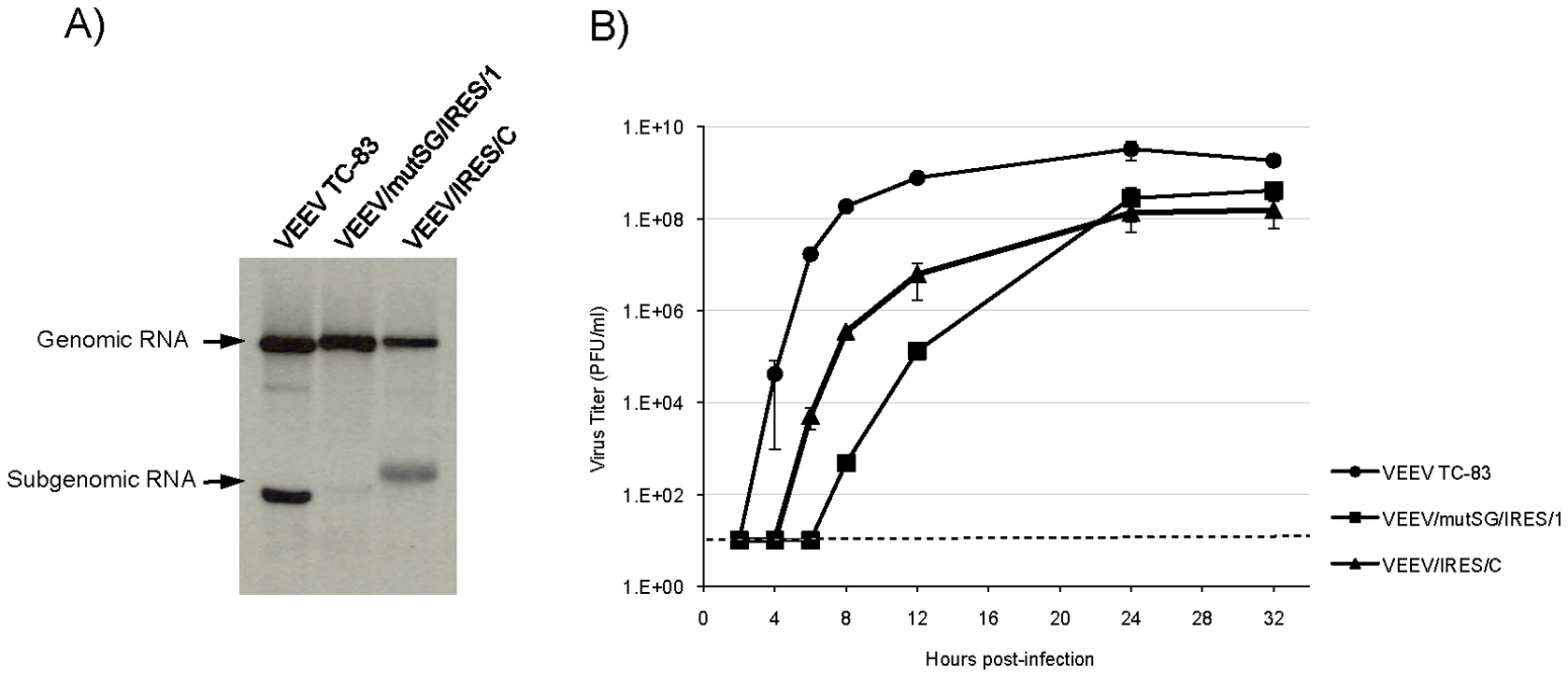
RNA synthesis and replication of VEEV TC-83 and TC-83/IRES constructs in BHK cells after electroporation. (A) Analysis of ^3^[H]-labeled viral RNA production and (B) viral replication after transfection of 4 µg of *in vitro*-synthesized RNA into BHK cells. Dark dashed line indicates the limit of detection for the experiment.

After SP6-driven *in vitro* RNA synthesis and electroporation into BHK-21 cells, production of viral RNAs (genomic and subgenomic) from the newly designed IRES-based vaccine candidate was confirmed *in vitro* (Fig. 2A). As previously shown, VEEV/mutSG/IRES/1 was incapable of producing subgenomic RNA due to the 13 point mutations introduced into the subgenomic promoter [22]. In VEEV/IRES/C, the subgenomic promoter was left intact, allowing efficient production of subgenomic RNA, which migrated more slowly than its TC-83 counterpart due to the introduction of the additional IRES sequence. However, it appeared that VEEV/IRES/C genomic RNA was produced at slightly lower levels compared to TC-83 and VEEV/mutSG/IRES/1. To assess the potential effect on viral replication, the production of infectious virus was monitored (Fig. 2B). As expected, both IRES constructs produced significantly less infectious virus than unmodified TC-83, with a difference of approximately 1.5 log_10_ at the peak of production; TC-83 titer reached 4.8×10^9^ PFU/ml at 24 h post-electroporation. Despite the difference in genomic RNA production, both IRES constructs reached their peak titer 24 h post-electroporation, with similar titers of 9×10^7^ PFU/ml for VEEV/mutSG/IRES/1 and 8×10^7^ PFU/ml for VEEV/IRES/C. Thus, the lower amount of genomic RNA produced by VEEV/IRES/C did not severely impair viral replication compared to VEEV/mutSG/IRES/1. Moreover, VEEV/IRES/C viral production was consistently detected ca. 2 hr earlier than that of VEEV/mutSG/IRES/1, and the latter exhibited significantly lower titers of production during the first 24 h post-electroporation.

### Replication in mammalian cells

**Figure 3:**
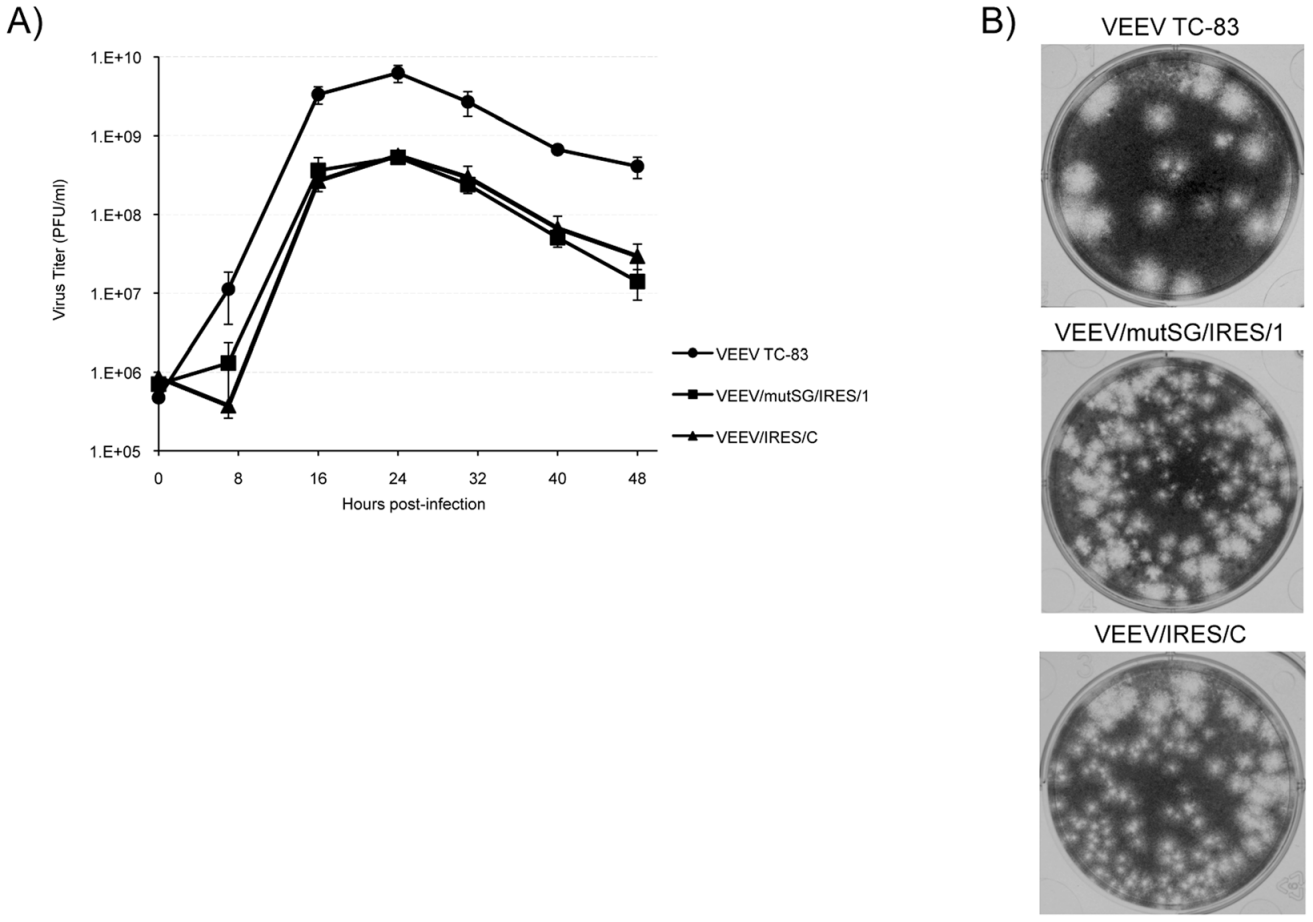
Replication of recombinant TC-83/IRES viruses and parental TC-83 on Vero cells. (A) Cells were infected with VEEV TC-83, VEEV/mutSG/IRES/1 and VEEV/IRES/C at an MOI of 0.1 then viral titers in collected supernatants over time were determined by plaque assay. Bars indicate standard deviation for duplicate infections. (B) Vero cells were fixed with 10% formaldehyde 48 h post-infection and stained with crystal violet to observed plaque size and morphology.

Viral replication following infection of Vero cells, an approved vaccine substrate, was also measured (Fig. 3A). Replication profiles were very similar to those obtained on BHK cells after electroporation, with an advantage of approximately 1 log for TC-83 compared to the IRES-modified strains, and a peak TC-83 titer of 6.2×10^9^ PFU/ml at 24 h post-infection, compared to 5.2×10^8^ and 5.3×10^8^ PFU/ml for VEEV/mutSG/IRES/1 and VEEV/IRES/C, respectively. Additionally, plaques produced under 0.4% agarose on Vero cells were visible as early as 24 h post-infection for TC-83, whereas 48 h of incubation was necessary for IRES-based viruses to produce visible plaques. This slower replication level was also correlated to the size of the plaques produced by IRES-based viruses on Vero cells (Fig. 3B). At 48 h post-infection, TC-83 produced 3–6 mm plaques whereas VEEV/mutSG/IRES/1 and VEEV/IRES/C plaques were 2–3 mm and 1.5–3 mm, respectively.

### Genetic and phenotypic stability *in vitro* and *in vivo*

**Figure 4:**
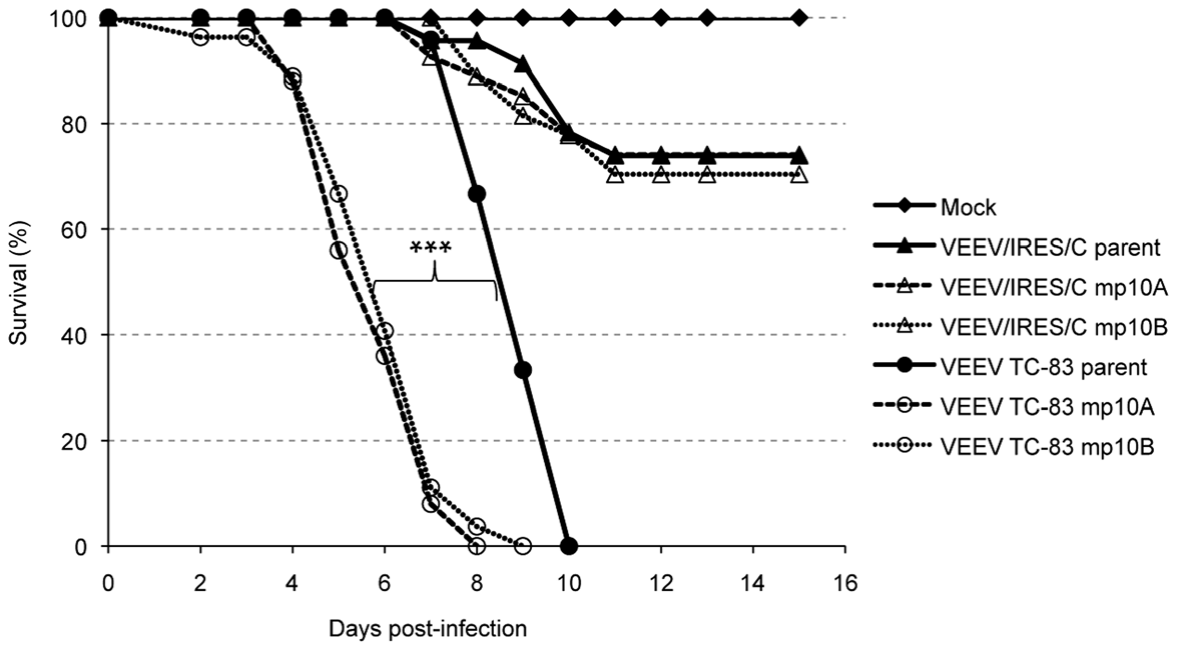
Survival in mice after infection with VEEV/IRES/C or VEEV TC-83 passaged *in-vivo*. Six-day-old CD1 pups received a 5×10^4^ PFU dose SC of viruses passaged 10 times in CD1 mice (mp10). Parent unpassaged VEEV TC-83 and VEEV/IRES/C were injected at the same dose as controls. Animals were monitored daily for survival for 14 days. No deaths occurred after day 11 post-infection. *** = P<0.0001.

VEEV/IRES/C was subjected to 5 serial passages in Vero cells or 10 serial passages in mouse brains. No discernible change was observed in plaque morphology after 5 serial passages *in vitro* or 10 passages *in vivo* (data not shown). Genetic stability was confirmed by full-genome sequencing of passaged viruses; no mutations were found in consensus sequences of Vero- or mouse-passaged viruses, aside from the deletion of one adenosine in a poly-A tract within the IRES itself, which appeared between passage 3 and 4 on Vero cells, and before passage 5 in mouse brains. No changes were detected in virulence for the mp10 VEEV/IRES/C compared to the parental strain (P = 0.95 for series A and P = 0.75 for series B) after SC injection of 6-day-old mice (Fig. 4), whereas a significant increase in virulence was observed for the mp10 TC-83 viruses compared to parental TC-83, as previously described [29].

### Replication in mosquito cells

**Figure 5:**
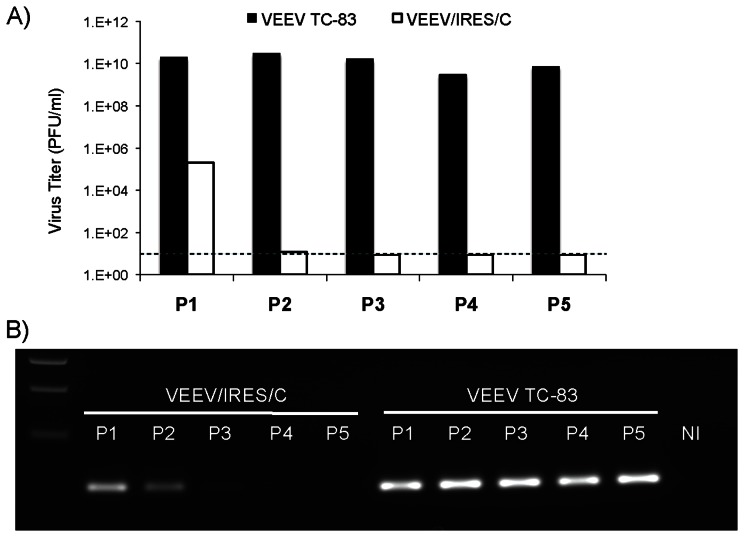
Replication of recombinant TC-83/IRES viruses in mosquito cells. (A) Five serial passages were performed on C6/36 cells at an initial MOI of 0.1 with VEEV/IRES/C, as well as VEEV TC-83 as a control. Supernatants were analyzed by plaque assay for detection of viral replication. Dark dashed line indicates the limit of detection for the experiment. (B) Supernatants were subjected to RT-PCR analysis to detect presence of viral RNA. Supernatant from non-infected cells (NI) was used as negative control.

To confirm its predicted inability to replicate in mosquito cells, VEEV/IRES/C was blind-passaged 5 times in C6/36 cells (along with TC-83 as a control). For each passage, supernatants were subjected to RT-PCR for viral RNA detection, and plaque assay for infectious virus. Virus and viral RNA were only detected in passages 1 and 2, presumably due to residual virions that were incompletely washed from the cells after the original inoculation (Fig. 5A and 5B). Indeed, the VEEV/IRES/C viral titer declined from 10^5^ PFU/ml in passage 1 to 10 PFU/ml after passage 2, along with weakening of the RT-PCR signal. No infectious virus or viral RNA was detected after 2 passages. Meanwhile, TC-83 virus consistently produced ca. 10^10^ PFU/ml, confirmed by the detection of viral RNA in supernatants for all 5 passages.

### Replication in mosquitoes

The predicted VEEV/IRES/C inefficiency of replication was also confirmed in live mosquitoes, and compared to TC-83. *Ae. aegypti* were allowed to feed on infectious blood meals containing 3×10^8^ PFU/ml of TC-83 or VEEV/IRES/C, and incubated for 10 days before being triturated and tested for the presence of infectious virus by detection of CPE on Vero cells. Fifty percent (24/48) homogenates from mosquitoes exposed to TC-83 produced detectable CPE, whereas none of the VEEV/IRES/C-exposed mosquitoes produced CPE after 10 days of incubation (Table 1). Because only 50% of the mosquitoes were found susceptible to TC-83, a second experiment was performed using a more permissive route of infection, intrathoracic injection. Using the highest dose achievable of 10^5^ PFU per mosquito (ca. 1 µl of a 10^8^ PFU/ml viral stock), 100% of mosquitoes injected with TC-83 became infected, whereas only 14/55 mosquitoes inoculated with VEEV/IRES/C produced CPE after incubation. Plaque assays performed on these homogenates revealed a mean titer of only 100 PFU/mosquito for the VEEV/IRES/C-infected mosquitoes, a titer incompatible with VEEV transmission by mosquitoes [30], [31], [32], [33], [34], [35], [36], [37], [38], [39]. In contrast, an average of 9×10^6^ PFU/mosquito were recorded in the TC-83-infected group. To determine if the presence of VEEV/IRES/C in mosquitoes 10 days after inoculation could have represented residual inoculum without replication, replicates of a viral suspension containing 10^6^ PFU/ml (VEEV TC-83 or VEEV/IRES/C) were incubated at 29°C for 10 days; these samples still contained on average 10^3^ PFU/ml after these 10 days, indicating that the CPE-positive mosquitoes injected with VEEV/IRES/C most likely contained only residual virus from the inoculum rather than supported active viral replication.

**Table 1:** Virus replication in *Ae. aegypti* mosquitoes.

	Artificial blood meal		Intrathoracic injection	
Vaccine Strain	VEEV TC-83	VEEV/IRES/C	VEEV TC-83	VEEV/IRES/C
**Inoculum titer** §	4×10^8^	7×10^8^	2×10^8^	3×10^8^
**Dose per mosquito**	N.A	N.A	ca. 10^5^ PFU	ca. 10^5^ PFU
**% CPE-positive (** ***n*** **)** *	50 (48)	0 (48)	100 (16)	25 (55)
**Homogenates Titer** #	N.D	N.D	2,6×10^7^	3×10^2^
**Titer per mosquito** ¶	N.D	N.D	ca. 9×10^6^ PFU	ca. 1×10^2^ PFU

§ PFU/ml.

* Cytopathic effects (CPE) detected on Vero cells 5 days after infection with homogenized-mosquito supernatants (*n*, sample size).

# Average virus titer in homogenized-mosquito supernatants found positive for CPE, in PFU/ml.

¶ Average virus titer in mosquitoes found positive for CPE.

### Attenuation in infant mice

**Figure 6:**
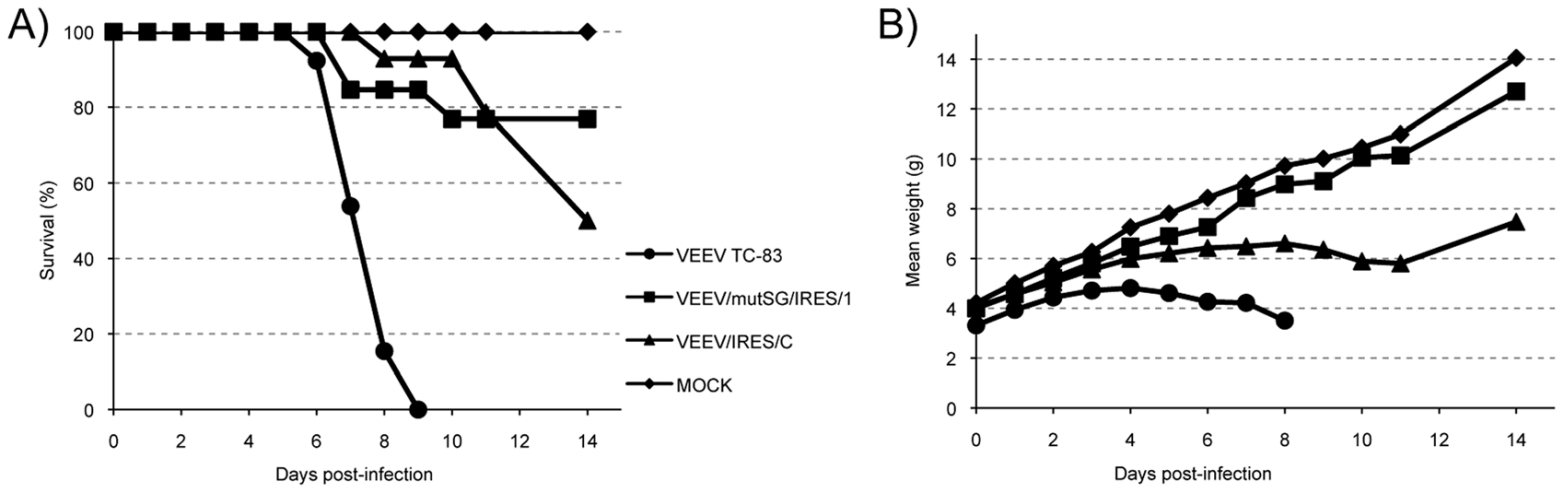
Virulence in mice after injection of VEEV TC-83 and IRES-based viruses. Six-day-old CD1 mice received 5×10^4^ PFU of indicated viruses SC, and were monitored 2 weeks for survival (A) and weight change (B). No deaths were recorded after day 14 post-infection.

Because TC-83 does not typically induce mortality in adult mice, an infant mouse model was used to compare virulence of the vaccine constructs. Cohorts of 6-day-old CD-1 mice were inoculated subcutaneously with 5×10^4^ PFU of TC-83, VEEV/mutSG/IRES/1 or VEEV/IRES/C and monitored for signs of illness, weight and survival. Another cohort of mice was inoculated with PBS as a negative control. As shown in Fig. 6A, while TC-83 induced 100% mortality by day 9 post-inoculation, the IRES-based viruses were both markedly attenuated (Logrank test, P<0.0001), with no significant difference observed between VEEV/mutSG/IRES/1 (76% survival) and VEEV/IRES/C (50% survival) at 14 days post-inoculation (P = 0.28). However, the mean weights recorded throughout the experiment indicated that growth of mice inoculated with VEEV/IRES/C was delayed compared to those inoculated with VEEV/mutSG/IRES/1 or PBS (P = 0.019 and P = 0.004 respectively, Fig. 6B), suggesting lesser attenuation of VEEV/IRES/C vaccine candidate compared to the previous IRES-based virus. However, the delayed growth in the VEEV/IRES/C was temporary and animals recovered in a few days, whereas no recovery was observed in the TC-83 group.

**Figure 7:**
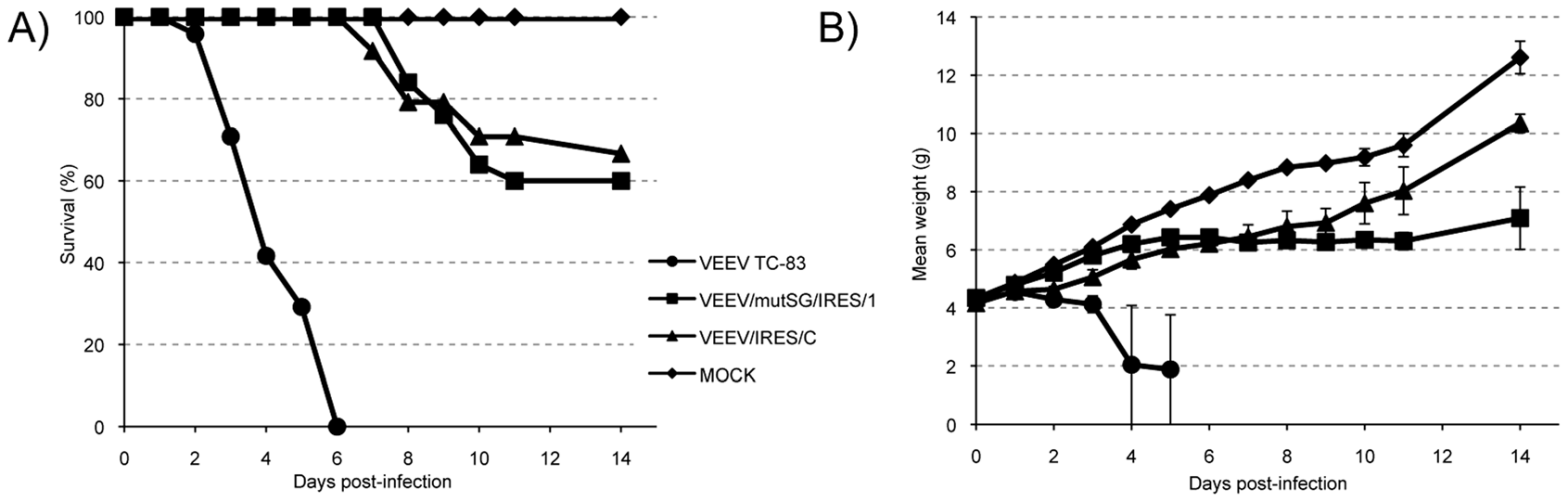
Neurovirulence in 6-day-old mice following IC injection of VEEV TC-83 and IRES-based viruses. Animals received 1×10^6^ PFU of indicated viruses, and were monitored for survival (A) and weight change (B) for 2 weeks, with no deaths recorded after day 14 post-infection.

To assess neurovirulence, six-day-old mice were inoculated intracranially with 1×10^6^ PFU of virus. Similar to that observed after subcutaneous inoculation, there was no significant difference between VEEV/mutSG/IRES/1 and VEEV/IRES/C cohorts in mortality, with 60% and 67% of the animals surviving, respectively (Logrank test, P = 0.45), whereas 100% mortality was observed at 6 days post-inoculation in the TC-83 group (P<0.0001, Fig. 7A). The mortality observed in the VEEV/mutSG/IRES/1 and VEEV/IRES/C groups was also delayed compared to TC-83. Nevertheless, animals inoculated with VEEV/mutSG/IRES/1 showed more signs of illness than animals inoculated with VEEV/IRES/C, illustrated by the observation of more delayed growth compared to the PBS group (P = 0.01, Fig. 7B) and neurological signs such as ataxia, paralysis and lethargy in most mice infected with VEEV/mutSG/IRES/1. Thus, in this model VEEV/IRES/C appeared to be less virulent than VEEV/mutSG/IRES/1.

### Immunogenicity and protection against VEEV challenge

**Figure 8:**
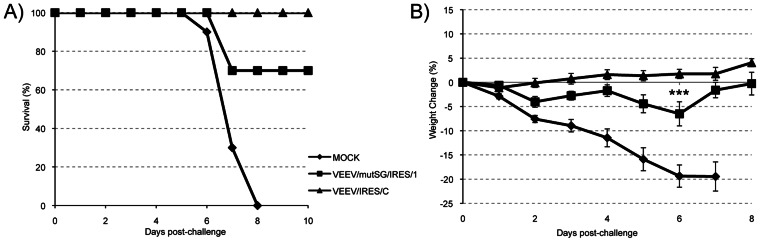
Protection against challenge following vaccination of infant mice. Six-day-old mice were inoculated SC with 5×10^4^ PFU of VEEV TC-83 or IRES-based viruses. Animals were challenged 6 weeks post-vaccination with 10^4^ PFU SC of VEEV IC strain 3908 and monitored daily for survival (A) and weight change (B), with no deaths recorded after day 8 post-challenge. *** = P<0.0001.

The ability of the new VEEV/IRES/C vaccine candidate to induce neutralizing antibodies and to protect against a lethal VEEV challenge was evaluated in neonatal and adult mouse models and compared to VEEV/mutSG/IRES/1 and TC-83. Animals that survived the single SC inoculation with VEEV/mutSG/IRES/1 and VEEV/IRES/C at 6 days of age were held for 6 weeks post-infection before sera were collected and tested by PRNT. Seroconversion was detected in 6 of 7 (85%) animals vaccinated with VEEV/IRES/C and in 6 of 10 (60%) animals vaccinated with VEEV/mutSG/IRES/1, with mean PRNT_80_ titers of 26±8 and 57±22, respectively (Table 2). Challenge was performed on these animals 3 weeks later with virulent VEEV strain 3908, a human isolate from the last major VEE epidemic [40], at a SC dose of 10^4^ PFU (ca. 10^4^ LD_50_). All sham-vaccinated animals died between days 6 and 8, whereas 30% mortality was recorded for the animals that received VEEV/mutSG/IRES/1, and all animals vaccinated with VEEV/IRES/C survived challenge (Fig. 8A). No weight loss was observed in the VEEV/IRES/C-vaccinated cohort after challenge, whereas the VEEV/mutSG/IRES/1- and sham-vaccinated animals lost an average of 6.5% and 19.4% of pre-challenge weight by day 6 post-challenge, respectively (Fig. 8B).

**Table 2:** Seroconversion of infant CD1 mice.

	Vaccine strain		
	VEEV/mutSG/IRES/1	VEEV/IRES/C	MOCK
**Fraction seroconversion**	6/10	6/7	0/10
**Mean PRNT_80_ Titer ± SD** * **^a^**	26±31	57±31	<20
**Mean PRNT_50_ Titer ± SD** * **^b^**	50±63	103±60	<20
**Protection after challenge** §	70%	100%	0%

* Reciprocal titer of serum capable of neutralizing ^a^80% or ^b^50% of TC-83 plaques (SD, standard deviation).

§ Challenge with 10^4^ PFU of VEEV IC strain 3908, 6 weeks post-vaccination.

**Figure 9:**
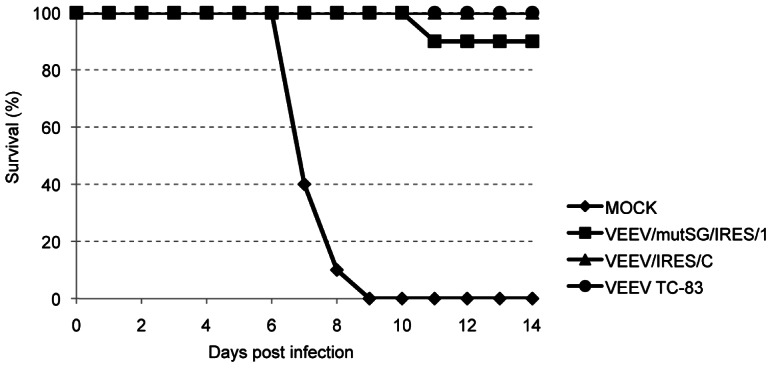
Survival following vaccination and challenge of adult mice. Five-week-old CD1 mice were vaccinated with 10^5^ PFU of VEEV TC-83 or IRES-based viruses. Challenge was performed 6 weeks post-vaccination by SC inoculation of 10^4^ PFU of VEEV IC strain 3908, with daily monitoring of animals. No deaths occurred after day 11 post-challenge.

In a second experiment, adult mice were vaccinated SC with a single dose of 10^5^ PFU of each vaccine strain. No viremia was detected in VEEV/mutSG/IRES/1- and VEEV/IRES/C-vaccinated groups at days 1 and 2 post-vaccination. In the TC-83-vaccinated group, 3 out of 5 animals were viremic on days 1 and 2 with mean titers of 2×10^3^ and 2×10^2^ PFU/ml, respectively. No significant weight changes were detected in any of the groups post-vaccination (data not shown). Animals were bled 2 months later and neutralizing antibody titers were determined. In the TC-83 vaccinated group, 100% of the animals seroconverted and PRNT titers all exceeded the endpoint of 1280. Although the titers in the IRES-recombinants vaccinated groups were lower than those in the TC-83 group, mean PRNT_80_ and PRNT_50_ titers were 2.5 times higher in the VEEV/IRES/C group (184±184 and 424±482, respectively) compared to VEEV/mutSG/IRES/1 group (74±98 and 160±195 respectively), with 80% seroconversion in VEEV/IRES/C-vaccinated animals and 70% in the VEEV/mutSG/IRES/1 cohort (Table 3). A challenge was performed 6 weeks post-vaccination with 10^4^ PFU of wild-type VEEV strain 3908. All sham-vaccinated animals died between days 7 and 9 post-challenge, whereas all animals vaccinated with VEEV TC-83 or VEEV/IRES/C were protected. One VEEV/mutSG/IRES/1-vaccinated animal died on day 10 post-challenge (Fig. 9). All sham-vaccinated animals had detectable viremia up to 4 days post-challenge, reaching an average of 1.3×10^7^ PFU/ml on day 3 (Table 4). In the VEEV/IRES/C-vaccinated group, viremia was recorded in 1, 3 and 1 animals out of 10 on days 1, 2 and 3 post-challenge, respectively, with average titers of 1×10^2^ PFU/ml on days 1 and 3, and 1×10^3^ PFU/ml on day 2. Challenge viremia was detected in 2 out of 10 animals vaccinated with VEEV/mutSG/IRES/1 on days 1 and 3, with average titers of 1×10^4^ PFU/ml and 1×10^2^ PFU/ml respectively. No virus was detected after challenge in animals vaccinated with TC-83 (Table 4). No significant difference was observed in weight change among the vaccinated groups (data not shown).

**Table 3:** Seroconversion of adult mice after vaccination.

	Vaccine Strain			
	VEEV/mutSG/IRES/1	VEEV/IRES/C	VEEV TC-83	MOCK
**Fraction seroconversion**	7/10	8/10	10/10	0/10
**Mean PRNT_80_ Titer ± SD** * **^a^**	74±98	184±184	≥1280	<20
**Mean PRNT_50_ Titer ± SD** * **^b^**	160±195	424±482	≥1280	<20
**Protection after challenge** §	90%	100%	100%	0%

* Reciprocal titer of serum capable of neutralizing ^a^ 80% or ^b^ 50% of TC-83 infection (SD, Standard Deviation).

§ Challenge with 10^4^ PFU of VEEV strain 3908, 6 weeks post-vaccination.

**Table 4:** Viremia in vaccinated adult mice after challenge.

	Vaccine Strain			
Days post-challenge*	VEEV/mutSG/IRES/1	VEEV/IRES/C	VEEV TC-83	MOCK
**1**	2/10§ (1×10^4^)#	1/10 (1×10^2^)	0/10 (-)	10/10 (8×10^5^)
**2**	0/10 (-)	3/10 (3×10^3^)	0/10 (-)	10/10 (7,7×10^5^)
**3**	2/10 (1×10^2^)	1/10 (1×10^2^)	0/10 (-)	10/10 (1,3×10^7^)
**4**	0/10 (-)	0/10 (-)	0/10 (-)	10/10 (1,5×10^4^)

* Days post-challenge with 10^4^ PFU of VEEV strain 3908, 6 weeks post-vaccination.

§ Fraction of animals with detectable viremia (Limit of detection 1×10^2^ PFU/ml).

# Mean viremia titer in serum, in PFU/ml.

## Discussion

Vaccines remain the best tools to control viral infectious diseases, for which there are few treatments available. Because they induce robust and often life-long protective immune responses, live-attenuated vaccines have been developed and used extensively for decades against viral diseases with remarkable successes [41]. Traditionally, these vaccines were derived empirically from wild-type virus strains by serial passages in animals or cell cultures. However, this approach often yields unpredictable results and poses safety concerns, including the risk of reversion to a wild-type phenotype, especially when the attenuation relies on a limited number of point mutations. VEEV vaccine strain TC-83 exemplifies this safety issue, as only 2 point mutations are responsible for its attenuation [14]. Probably as a consequence, TC-83 is reactogenic in many human vaccinees, which has prevented its licensure [21], [42], [43]. However, TC-83 has been studied extensively and licensed in several countries for veterinary use, for which it is sufficiently attenuated and immunogenic [42], [44]. Thus, it represents a suitable backbone to develop a safer and more attenuated VEEV vaccine.

In a previous study, a recombinant TC-83 virus was developed by placing the expression of the viral structural proteins under the vertebrate-restricted translation control of the EMCV IRES, which does not efficiently drive protein expression in mosquito cells [22]. This strategy resulted in 2 critical improvements over unmodified TC-83: 1) the IRES-recombinant TC-83 was more attenuated and thus potentially less reactogenic, and; 2) it was incapable of replication in mosquitoes, which dramatically reduces the risk of initiating a mosquito-vertebrate amplification cycle from a vaccinated and viremic equid, and the subsequent potential for reversion to virulence. However, this first generation of IRES-based TC-83 vaccine did not induce detectable neutralizing antibodies in the NIH Swiss mice model and failed to protect 100% of challenged animals. As suggested previously [45], the low level of structural protein expression observed for the IRES-recombinant TC-83 virus could explain its poor immunogenicity, as critical B cell epitopes are located in the surface glycoproteins E1 and E2 [46], [47].

To retain the benefits of the first generation of IRES-based TC-83 vaccine while increasing the expression of the glycoproteins E1 and E2, we placed the capsid gene at the 3′ end of the structural protein open reading frame and under EMCV IRES control. Expression of the surface glycoproteins E1 and E2 was left under the control of the viral subgenomic promoter in a cap-dependent manner, as in the parental TC-83. As in the first TC-83 IRES-recombinant version, the deletion of the IRES sequence would make VEEV/IRES/C non-viable because the capsid gene could not be translated from the subgenomic RNA. VEEV/IRES/C was efficiently rescued and produced high titers on Vero cells, an acceptable substrate for vaccine production, making VEEV/IRES/C a vaccine candidate feasible to produce to large scale.

By comparing the new VEEV/IRES/C to the previous IRES-based TC-83 vaccine candidate, VEEV/mutSG/IRES/1, and the parental strain TC-83, we demonstrated that placing the capsid protein under IRES control while leaving the envelope glycoproteins under the subgenomic promoter control did not increase viral yields *in vitro* or greatly increase virulence in the mouse model. This could simply reflect an unbalanced ratio of capsid versus glycoproteins, which would not allow highly efficient encapsidation and release of viral particles. Overall, VEEV/IRES/C exhibited a similar attenuation profile compared to VEEV/mutSG/IRES/1 and markedly greater attenuation compared to VEEV TC-83. Additional studies in adult mice and eventually in non-human primates and horses will be necessary to link the increased attenuation of this virus to a decreased reactogenicity. Further investigating the pathogenesis of VEEV/IRES/C in terms of tissue tropism will also be needed to support its further development.

In terms of environmental safety, we also demonstrated that the consensus genome sequence of VEEV/IRES/C was stable after serial passages *in vitro* or *in vivo*, which translated to phenotypic stability *in vivo* with no significant change in virulence. In contrast, TC-83 underwent a rapid and significant increase in virulence after mouse passages, presumably reflecting its unstable attenuation based on only 2 point mutations [14]. Kenney *et al.* showed similar results and the increased TC-83 virulence was associated with a mixture of mutants, suggesting that a complex quasispecies population determined the virulence phenotype [29]. VEEV/IRES/C was also incapable of replicating in mosquito cells in vitro. Although we found small amounts of residual virus in a small proportion of IT-injected mosquitoes after 10 days of incubation, the low titers suggested residual viral inoculum rather than productive viral replication. In parallel, we showed that no mosquitoes were infected with VEEV/IRES/C after exposure to a large oral dose that far exceeded the 3 log_10_/ml detected in humans or 3.5 log_10_/ml detected in horses vaccinated with TC-83 [21], [42]. Thus, the inability of VEEV/IRES/C to replicate in mosquitoes offers a major advantage, even compared to another live-attenuated VEEV vaccine candidate, strain V3625, which is able to replicate to high titers in mosquitoes [48], [49].

Immunogenicity and efficacy were assessed after vaccination of infant and adult mice. In both models, VEEV/IRES/C appeared to be more potent at inducing a neutralizing antibody response compared to VEEV/mutSG/IRES/1. Although the PRNT titers were lower for VEEV/IRES/C compared to TC-83, all animals were protected from lethal VEEV challenge, whereas VEEV/mutSG/IRES/1 failed to do so. Moreover, animals that survived challenge after VEEV/mutSG/IRES/1 vaccination showed weight loss, where no signs of disease were observed in the VEEV/IRES/C-vaccinated group either after vaccination or challenge. Volkova *et al.* showed that adult NIH Swiss mice vaccinated with ca. 10^5^ PFU of VEEV/mutSG/IRES/1 virus failed to develop detectable neutralizing antibodies and only 80% of the vaccinated animals were protected against a challenge with 10^4^ PFU of wild-type VEEV strain 3908, versus 100% protection obtained with TC-83 [22]. In similar experiments with NIH Swiss mice, VEEV/IRES/C induced neutralizing antibody response and were fully protected against lethal challenge with VEEV 3908 (data not published). These results support the greater immunogenicity of VEEV/IRES/C compared to the first IRES-based TC-83 vaccine candidate.

These promising observations need to be confirmed by more extensive exploration of the immune response induced by VEEV/IRES/C, by testing different vaccine doses, and by evaluating the duration of immunity and protection against aerosol exposure. It would also be interesting to investigate the innate immune response induced, as it was previously shown that more type I interferon (IFN) was produced by cells infected with VEEV/mutSG/IRES/1 compared to TC-83 [22]. The capsid proteins of VEEV (and the closely related eastern equine encephalitis virus), involved indirectly in the antagonism of cellular antiviral responses through cellular transcription shutoff [50], [51], [52], remains under the control of the IRES in VEEV/IRES/C, which could imply a lower level of its expression and thus a reduced inhibition of the cellular antiviral response, including type I IFN. If this pattern is confirmed in the course of VEEV/IRES/C infection, it could potentially influence the nature and quality of the adaptive immune response, which is regulated by the innate immune response [53], [54], [55]. Moreover, neutralizing antibodies are not absolutely required for protection against VEEV challenge [22], [56], a finding supported by our data showing the survival of some challenged animals without detectable neutralizing antibodies. These observations suggest a significant role of the cellular adaptive immune compartments in protection against VEEV infection. Paessler *et al.* also demonstrated that T-cells alone protected against encephalitis following VEEV infection [56]. Thus, although the humoral response to VEEV/IRES/C appears to be lower than that induced by TC-83, the cellular compartment should also be evaluated.

In conclusion, we demonstrated that this novel, IRES-based TC-83 recombinant virus is superior to TC-83 in attenuation yet provides equivalent protection in a mouse model. Its inability to infect mosquitoes increases its safety by reducing the potential for natural spread after vaccination followed by reversion, which could lead to the initiation of an epidemic. Finally, this study also demonstrates that the IRES can be positioned alternatively to achieve the optimal balance between attenuation and immunogenicity, and along with other studies performed with chikungunya and eastern equine encephalitis viruses [28], [45], [57], further validate the IRES attenuation strategy as an effective and predictable approach for vaccine development against other alphaviruses constantly threatening developing countries.
